# Neuro-Endocrine Networks Controlling Immune System in Health and Disease

**DOI:** 10.3389/fimmu.2014.00143

**Published:** 2014-04-07

**Authors:** Claudio Procaccini, Valentina Pucino, Veronica De Rosa, Gianni Marone, Giuseppe Matarese

**Affiliations:** ^1^Laboratorio di Immunologia, Istituto di Endocrinologia e Oncologia Sperimentale, Consiglio Nazionale delle Ricerche, Napoli, Italy; ^2^Dipartimento di Scienze Mediche Traslazionali, Università di Napoli “Federico II”, Napoli, Italy; ^3^Unità di Neuroimmunologia, IRCCS Fondazione Santa Lucia, Roma, Italy; ^4^Centro Interdipartimentale di Ricerca in Scienze Immunologiche di Base e Cliniche, Università di Napoli “Federico II”, Napoli, Italy; ^5^Dipartimento di Medicina e Chirurgia, Università degli Studi di Salerno, Salerno, Italy; ^6^IRCCS Multimedica, Milano, Italy

**Keywords:** neuro-immune modulation, leptin, autoimmunity, MS, metabolism

## Abstract

The nervous and immune systems have long been considered as compartments that perform separate and different functions. However, recent clinical, epidemiological, and experimental data have suggested that the pathogenesis of several immune-mediated disorders, such as multiple sclerosis (MS), might involve factors, hormones, and neural mediators that link the immune and nervous system. These molecules are members of the same superfamily, which allow the mutual and bi-directional neural–immune interaction. More recently, the discovery of leptin, one of the most abundant adipocyte-derived hormones that control food intake and metabolism, has suggested that nutritional/metabolic status, acting at central level, can control immune self-tolerance, since it promotes experimental autoimmune encephalomyelitis, an animal model of MS. Here, we summarize the most recent advances and the key players linking the central nervous system, immune tolerance, and the metabolic status. Understanding this coordinated interaction may pave the way for novel therapeutic approaches to increase host defense and suppress immune-mediated disorders.

## Introduction

The central nervous system (CNS) has been considered for a long time, a privileged organ thanks to its inability to start an immune response against antigens. However, accumulating evidence has shown the presence of a mutual interaction between the immune system and CNS in physiological as well as in pathological conditions. Indeed, the CNS displays a well-organized innate immune reaction to infection and immune cells express on their surface several receptors for different neurotransmitters, which allow the brain to modulate the immune system functions and keep the homeostasis of the whole body in an appropriate manner, by responding to environmental changes (1–6). Moreover, immune cells can also synthesize and secrete several hormones with immunomodulatory properties (2, 7, 8) that can reduce or inhibit any exacerbated inflammatory response; for instance, the lymphocytes (9) and macrophages (10) produce the endogenous opioid peptides and catecholamines such as norepinephrine (NE) and epinephrine (E) (11). Furthermore, human lymphocytes secrete the growth hormone (GH) (12) and monocytes secrete the brain-derived neurotrophic factor (BDNF), whose expression is up-regulated by flogistic mediators such as TNF-α and IL-6 (13, 14). Recently, it has been shown that circulating LPS is able to induce the transcription of genes encoding for CD14 (its receptor) and toll-like receptor 2, as well as a wide variety of pro-inflammatory molecules in circum-ventricular organs (CVOs) (15).

A delayed response to LPS occurs in cells located at the edge of the CVOs and in microglia throughout the CNS. Pathogens can then induce the activation of the innate arm of the immune system in neuronal tissue, without having direct access to it.

## Immune-Surveillance in the Control of CNS

Although the CNS lacks lymphatics, it expresses major histocompatibility complex (MHC) molecules and the blood–brain barrier (BBB), and the blood–cerebrospinal fluid (CSF) are able to ensure protection of CNS by the diffusion of infectious agents (16). Conversely, alteration of immunity is often associated with cerebral infections. In physiological conditions, the immune system monitors the integrity of the brain and spinal cord (immune-surveillance), in order to highlight any inflammatory mediators resulting from infection and damage. In this context, a key role in the control of immune-surveillance is played by the resident microglia and immune cells (16). Indeed microglial cells are able to activate the adaptive immune system, when required, and these glia cells are in turn modulated by endogenous mechanisms, thus confirming the tune control of immune system in the CNS (17). Microglia secrete neurotrophins, such as nerve growth factor (NGF), able to sustain neuronal and macroglial survival and growth. In addition to microglia, peripheral immune cells can reach the inflammatory site in the CNS, through mechanisms similar to those observed in peripheral organs. T cells travel into the CNS through transient interactions with CNS endothelium, which expresses cell adhesion molecules, moreover other immune cells (macrophages and dendritic cells) are located at the interface between the blood and brain, where they can promote antigen presentation and a powerful inflammatory response (18). Non-activated microglia express low levels of HLA-DR in the healthy human brain and MHC-II molecules (MHC-II, CD80, CD86, CD40, CD11a) in the rodent brain, thus suggesting their antigen presentation capability (19–24).

Recent evidence has revealed that T cells can be found in the CSF of healthy individuals, indicating that these cells can reach the CNS through the choroid plexus and meninges (25, 26). They have been characterized as CD4^+^CD45RA^−^CD27^+^CD69^+^ activated central memory T cells (27, 28), which expressed high levels of CCR7, CXCR3, and L-selectin (29, 30) and are located in brain areas, which lack of tight junctions in the BBB (31).

In conclusion, the fact that endogenous factors (such as trauma or immuno-suppressive agents) may cause alteration in the migratory capacity of immune cells in the CNS, leading to an uncontrolled proliferation of infectious agents with consequent occurrence of neurological complications, would indicate a clear and unambiguous key role of immune system in the “immunosurveillance” of the CNS.

## The Neural and Immune System Communication

### The autonomic nervous system

The control of inflammation is realized by two major mechanisms: self-controlling immune mechanisms and brain-derived immunoregulatory output. The CNS regulates immune function, inflammation, and pathogens responses against host tissues, through the production of inhibitory cytokines, hormones, and other soluble molecules able to signal to the brain, which in turn exerts strong regulatory effects on the immune response (5, 32). Brain immunoregulatory action is mediated by the autonomic nervous system, through sympathetic and vagus nerve innervation. Recent evidence has reported that afferent neurons express receptors for several pro-inflammatory cytokines, such as tumor necrosis factor (TNF), IL-1, activating neural reflex circuits that regulates acute and chronic immune responses (5). A prototypical example of neural circuit is the inflammatory reflex mediated by the vagus nerve and the α7 subunit of the nicotinic acetylcholine receptor (α7 nAChR) expressed on immune cells (33).

**Figure 1:**
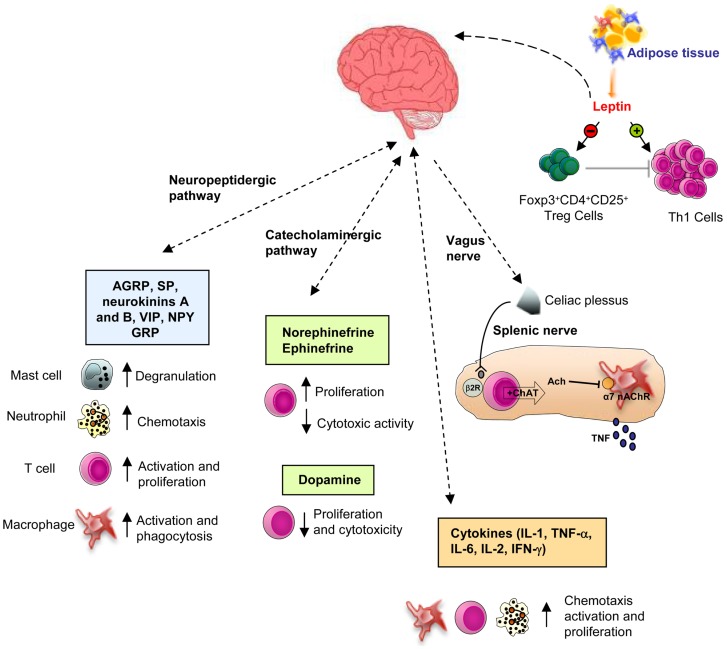
**Schematic representation of the CNS-immune system crosstalk**. There are bi-directional circuits linking CNS and immune system. The CNS can communicate with the immune system to modulate its activity, through different ways: through the autonomic nervous system (via the sympathetic and vagus nerve innervation, see the text for deeper details), the catecholaminergic pathway, or the neuropeptides and hormones release. In this context, leptin modulates immune system, by increasing the activation of T cells and decreasing Treg cells functions, thus representing a key player in the susceptibility to immune-mediated disorders (β2R, β2 receptor; α7 nAChR, α7 subunit of the nicotinic acetylcholine receptor).

Vagus nerve activation determines NE release from splenic neurons, which through the binding to β2 adrenergic receptor expressed on splenic T cells, favors choline acetyltransferase stimulation with consequent acetylcholine production (34) (Figure 1). T cell receptor (TCR)-mediated stimulation of splenic T cells significantly enhances their ability to produce acetylcholine, which binds to α7 nAChR expressed on macrophages resident in the red pulp and marginal zone of the spleen (35), thus suppressing NF-κB activity and consequently reducing cytokine synthesis (33, 35) (Figure 1). Activation of this pathway by electrical stimulation of the vagus nerve or administration of α7 selective agonists improves inflammation and survival in different clinical conditions (34). Moreover, confirming the essential role of T cells for vagus nerve action in the inhibition of cytokine release, it has been recently shown that the inflammatory reflex is impaired in nude mice, (34) and the adoptive transfer of T cells, which secrete enough amount of acetylcholine, since they express choline acetyltransferase, is able to revert this phenomenon, recovering the inflammatory reflex in these mice.

All these data have been confirmed also in humans; indeed patients with autoimmune disease and non-resolving inflammation display impaired vagus nerve signaling, which favors the progression of inflammation (32), whereas vagus nerve stimulation is able to attenuate leukocytes migration into the joints of synovitis affected patients (36). In line with this evidence, α7 nAChR deficient mice have increased synovial inflammation when compared to their littermate controls in a model of collagen-induced arthritis (37, 38). Treatment with α7 nAChR agonists or electrical vagus nerve stimulation significantly decreases arthritis in wild-type (WT) mice with collagen-induced arthritis. Finally, diet can also influence the inflammatory reflex; indeed dietary consumption of fish oil, significantly enhances the vagus nerve stimulation, favoring resolution of inflammation (39). On the other hand, in condition of obesity, where there is an inappropriate energy deposit and expenditure, leading to low grade inflammation and metabolic disease, an impaired vagus nerve activity has been found (40).

### The catecholaminergic pathway

Catecholamines [i.e., epinephrine, NE, dopamine (DA)] can regulate several functions of the immune system activities, such as proliferation, cytolytic activity, cytokine and antibody release, and chemotaxis, by interacting with adrenoreceptors expressed on lymphoid organs and immune cells. In particular, it has been demonstrated that NE and beta-adrenergic agonists are able to inhibit cytotoxic activity and increase lymphocytes proliferation (41–43). At the same time, high amount of DA was found to significantly inhibit the *in vitro* proliferative response and cytotoxic activity of T cells (44) (Figure 1). Moreover, it has been recently reported an enhanced proliferation and an impaired secretion of interferon-γ (IFN-γ) in the spleen of mice treated with DA (45). Recent evidence shows an important role of the hypothalamic–pituitary–adrenal (HPA) axis also in the bi-directional comunication between the brain and the immune system. GH and prolactin (PRL) are known to modulate immune responses (45–48). Indeed, several authors have shown that human GH significantly antagonizes the dexamethasone-induced inhibition of human T cell proliferation (46, 47). Moreover, secretion of PRL sustains antibody production and cell-mediated immune functions and therefore its inhibition increases the susceptibility to infectious diseases (47, 49). Glucocorticoids also exert several immunomodulant effects, such as the enhancement of T cells proliferation and survival (50, 51), and in physiological doses, the shift in cytokine secretion from a Th1 toward a Th2 phenotype (52, 53).

### The peptidergic pathway: Neuropeptides

Recent evidence suggests a key role of the neuropeptidergic pathway in the control of immune system (54, 55). Activation of nociceptors leads to local axon reflexes through the release of neuropeptides [i.e., calcitonin gene-related peptide (CGRP), substance P (SP), adrenomedullin, neurokinins A and B, vasoactive intestinal peptide (VIP), neuropeptide Y (NPY), and gastrin releasing peptide (GRP), etc.], which locally recruit and activate both innate and adaptive immune cells. More specifically, it has been shown that these mediators sustain chemotaxis and activation of neutrophils, macrophages, lymphocytes, and mast cells, increase the presenting capability of antigen presenting cells (APCs) and stimulate signaling to vascular endothelial cells, enhancing the recruitment of inflammatory leukocytes (55, 56) (Figure 1).

Another possible way of communication between immune cells and nociceptor neurons is also mediated by cytokine release. Indeed, sensory neurons display several cytokine receptors such as IL-1β receptor (IL-1βR) and TNF-α receptor (TNF-αR), which are able to recognize factors secreted by immune cells (i.e., IL-1β, TNF-α, NGF). They also express danger-associated molecular pattern (DAMP) receptors, toll-like receptors (TLRs), pathogen-associated molecular patterns (PAMPs), which recognize exogenous environmental signals (i.e., heat, acidity, chemicals, bacteria, viruses) or endogenous danger signals (i.e., ATP concentration, uric acid, hydroxynonenals) (56, 57), enhancing T cell functions (proliferation, cytokine secretion, and adhesion molecules expression) and thus representing a relevant player in CNS–immune system crosstalk in normal and pathophysiological conditions (58–61). In activated macrophages, VIP inhibits the expression of pro-inflammatory cytokines and chemokines (62–64), sustaining the differentiation of CD4^+^ T cells in Th2 cells and promoting their proliferation and/or survival (64, 65). Among the other neuropeptides, several functions of the cellular immune system have been shown to be regulated by NPY, SP, and related-agouti protein (AgRP) (66). NPY is a neuropeptide that increases food intake and storage of energy as fat but it is also able to modulate lymphocytes proliferation, NK activity, and interleukin-2 (IL-2) and TNF-α release (67). SP stimulates lymphocyte migration, proliferation, and IgA secretion and promotes phagocytosis and chemotaxis in innate immune cells, during inflammation (68). On the other hand, AgRP is co-expressed with NPY and works by increasing appetite and decreasing metabolism and energy expenditure. Hypothalamic AgRP neurons are mandatory for feeding and survival (69, 70) and they mediate effects of the histone deacetylase, Sirt1, on energy metabolism (71, 72). Recently, it has been shown that these neurons are involved in the regulation of adaptive immune responses. Indeed, knockdown of Sirt1 in Agrp neurons induce a pro-inflammatory state, characterized by a decrease in regulatory T cell functions with consequent increase of effector T cell activity, which determines an increased autoimmune disease susceptibility (73). This finding together with a recent paper by Luquet’s group (74) confirms the notion that the sympathetic nervous system may play a central role in mediating the effect of impaired function of AgRP neurons on immune system activity.

### Cytokines-related pathway

It is becoming well accepted that products of the immune system (cytokines) can signal the brain that infection has occurred. This cytokine-to-brain communication can result in marked alterations in brain function and behavior. In general, cytokines may traffic to the CNS at sites where the BBB is absent (75, 76), by carrier-mediated transport mechanisms, or by generating central mediators altering the permeability of the BBB to other substances (5, 77). Cytokines may also act directly on the CNS, by stimulating peripheral afferent neurons (5, 78). Indeed, peripherally generated cytokines can stimulate vagus nerve, which represents another very important pathway through which signals reach the brain (79). Several cytokines such as IL-1, IL-2, IL-6, IFN-γ, and TNF-α can regulate the activation of the HPA axis and are also influenced by glucocorticoid secretion (52, 80). IL-1 is one of the most studied cytokines linking immunological activation with the brain functions (81–83). Indeed, IL-1 has been shown to influence hypothalamic neurosecretory activity by stimulating CRH release by hypothalamic CRH neurons, and to enhance the turnover of NE in the hypothalamus (84, 85). IL-1 is also produced by several type of cells resident in the CNS, including astrocytes and microglia (86, 87) and IL-1 receptors have been identified in different brain areas, such as hippocampus and the dorsal raphe nucleus (88, 89). Furthermore, mRNA for IL-1α and TNF-α has been demonstrated in anterior pituitary cells (90, 91), which secrete IL-6 as well (91). IL-1 has been shown to be pivotal for the recruitment of leukocytes across the BBB. Indeed, recent studies have demonstrated that intracerebroventricular injection of IL-1β as well as IFN-γ and TNF-α induce neutrophils and leukocytes infiltration into the brain tissue (92), in a mouse model of experimental autoimmune encephalomyelitis (EAE) (93), by increasing the production of P-selectin on brain endothelial cells (94). In addition, also receptors for IL-2 were found in specific brain areas such as the hippocampal formation (95, 96) and it has been recently shown that that IL-2 deficiency results in altered septal and hippocampal structure, associated with changes in neurotrophins production (97).

## Leptin: At the Crossroad between CNS and Immune System Function

Leptin, the product of the obese (*ob*) gene, has been recently recognized as one of the most studied molecule linking CNS, nutrition, metabolism, and immune homeostasis (98). Leptin is mainly produced by the adipose tissue in proportion to the body fat mass and also by tissues such as the stomach, skeletal muscle, and placenta (98). At central level, this hormone regulates food intake, bone mass homeostasis, autonomic nervous system outflow, and the secretion of HPA hormones (98). Originally, leptin has been identified as the hormone responsible for the regulation of the balance between food intake and energy expenditure, being able to signal to the brain any changes in stored energy. However recent evidence has indicated that leptin is much more than a “fat-o-stat” sensor (99–101); indeed, leptin-deficient (*ob/ob*) and leptin-receptor-deficient (*db/db*) mice are not only strongly obese, but they also display several alterations, due to the effects of leptin on reproduction (102), hematopoiesis (103), angiogenesis (104), metabolism of bone (105), lipids and glucose (98) and, more importantly, innate and adaptive immunity (106, 107).

### Leptin and immune system regulation

Leptin has a well-established role in the modulation and regulation of innate immunity. Leptin increases phagocytic activity (108) and cytokine secretion (i.e., TNF-α, IL-6, and IL-12) (109, 110) in monocytes and macrophages, up-regulating the expression of activation markers, such as CD25 [α-chain of IL-2 receptor (IL-2R)], CD71 (transferring receptor), CD69, and CD38. Moreover, leptin can stimulate neutrophils chemotaxis and the oxidative burst (111, 112) and sustain proliferation, development, differentiation, activation, and lytic activity of NK cells, through the increase in perforin and IL-2 secretion (113). On the other hand, leptin exerts its effects also on adaptive immunity. Indeed, leptin modulates proliferation and cytokine production by both human naive (CD45RA) and memory (CD45RO) CD4^+^ T. On naïve T cells leptin promotes the proliferation and IL-2 secretion, whereas, on memory T cells, it promotes the switch toward T helper (Th)1-cell phenotype by increasing the secretion of pro-inflammatory cytokines such as IFN-γ and TNF-α (99). Leptin also supports immune cells migration to inflammatory sites, through the induction of IFN-γ production and the expression of adhesion molecules, such as intercellular adhesion molecule-1 (ICAM-1, CD54) and very late antigen-2 (VLA-2, CD49b) on CD4^+^ T cells.

Recent evidence indicates that leptin inhibits thymic T cells apoptosis, thus supporting their generation, maturation, and survival (114). Indeed, DTH responses and thymic atrophy have been shown to be decreased after acute caloric deprivation and serum leptin reduction; these conditions were restored by leptin treatment (114). Moreover, leptin can negatively modulate the expansion of human natural Foxp3^+^CD4^+^CD25^high^ regulatory T cells (nTregs) (115) (Figure 1), a cellular subset, which suppress autoreactive response mediated by CD4^+^25^−^ T (Teffs) cells. Treg cells produce leptin and express high levels of leptin receptor (ObR) (114). *In vitro* neutralization with anti-leptin monoclonal antibody (mAb) plus anti-CD3/CD28 stimulation causes Treg cells proliferation (115). This mechanism is mainly due to the down-regulation of the cyclin-dependent kinase inhibitor p27^kip1^ and the phosphorylation of the extracellular-related kinases 1/2 (ERK1/2), pivotal molecular pathways in the Treg cells activation and anergy (115). Moreover, an increased Treg cells proliferation has been observed in leptin- and ObR-deficient mice. Recently, it has been shown that leptin can potentiate the mTOR pathway activation, thus inhibiting rapamycin-induced proliferation of Tregs. In physiological circumstances, Tregs secreted leptin, which in turn activated mTOR pathway, a condition which sustains their state of hyporesponsiveness and anergy. Accordingly, Tregs from *db/db* mice had a reduced mTOR activity and enhanced proliferation compared with that of WT Tregs (116). Experiments by the same group have shown that leptin activates mTOR pathway also in Teffs, thus causing a defined cellular, biochemical, and transcriptional modification that determines the outcome of their responses, both *in vitro* and *in vivo*. Indeed, the blockade of leptin/leptin receptor signaling, induced by genetic means or by starvation, leads to impaired mTOR activity, which in turn inhibits the proliferation of Teffs (100). Taking together, these data suggest that the leptin-mTOR axis sets the threshold for the responsiveness of Treg and Teff cells, confirming that this pathway might integrate cellular energy status with metabolic-related signaling in Treg/Teff that use this information to control immune tolerance.

### Leptin and autoimmunity

Recent evidence indicates that leptin promotes pro-inflammatory cytokine secretion, thus enhancing immune responses in autoimmune disorders. In several autoimmune diseases, such as rheumatoid arthritis (RA), high serum leptin levels have been found, while, on the contrary, fasting, which associates with a marked decrease in serum leptin amount and a shift toward Th2-type cytokine secretion, improves clinical disease activity in RA patients (117). In line with these findings, another suggestion on the involvement of leptin signaling in the modulation of antigen-induced arthritis comes from studies showing that leptin-or leptin receptor (LepR)-deficiency protects mice from the development of autoimmune arthritis, after immunization with methylated bovine serum albumin (BSA) into knee joints, as these genetic conditions associate with decreased antigen-specific T cell proliferative responses (118). Recently, it has been reported that Th17 cell frequency is reduced in *ob/ob* mice and that the administration of leptin to *ob/ob* mice restore Th17 cell numbers to values comparable to those found in WT animals. Leptin promotes Th17 responses in normal human CD4^+^ T cells and in (NZB × NZW) F1 lupus-prone mice, by inducing RORγ transcription, whereas, on the contrary, its neutralization in those autoimmune-prone mice inhibits Th17 responses (119).

Leptin deficiency has been also associated with protection toward other inflammatory disease such as the experimentally induced glomerulonephritis, which is an immune-complex-mediated disorder (120). More specifically, studies from Lord’s group have shown that the renal protection observed in *ob/ob* mice has to be ascribed to a reduced glomerular-crescent formation and to an impaired macrophage recruitment in the site of inflammation (120). The reduced T cell proliferative profile and the altered humoral responses to sheep IgG, further support the authors’s hypothesis of consistent defects in innate and adaptive immune responses that can be considered crucial factors at the base of the protection to glomerulonephritis development in leptin-deficient mice.

Leptin has also been linked to spontaneous autoimmune disease such as Type 1 Diabetes (T1D) in the non-obese diabetic (NOD) mice. Indeed, this cytokine-like hormone accelerates the disease onset and progression by stimulating destruction of pancreatic β-cells by autoreactive T cells, which are further sustained to produce IFN-γ by leptin treatment (121).

Another indication for the important role of leptin in autoimmunity is the sexual dimorphism of serum leptin levels; indeed women display serum leptin levels two to three times higher than those observed in age- and BMI-matched men, and moreover, they are more prone to develop autoimmune diseases such as multiple sclerosis (MS), RA, or systemic lupus erythematosus, thus suggesting that leptin could favor the predisposition of females to this kind of disorders (122, 123).

Recent clinical studies on autoimmune disease patients demonstrate that high serum leptin levels may play a causal role in the disease progression, as previously mentioned, but at the same time might be utilized as a diagnostic marker for novel clinical application (122).

### Role of leptin in the pathogenesis of multiple sclerosis

Multiple sclerosis is an autoimmune disorder of the CNS, in which T cells specifically recognize myelin antigens and induce tissue damage, leading to lesion evolvement in the CNS with subsequent demyelination and axonal injury (124). Clinically, this disorder may present as relapsing-remitting type of MS (RRMS) (85%) or it may convert over time to a secondary chronic progressive type of MS (SP-MS). About 15% of cases present with a primary progressive disease course (PP-MS) and only few patients display a progressive relapsing MS disease course (PR-MS) with fast progression of the disease (125). In Europe and North America, the incidence is about 6/100,000/year and the prevalence 1/1000. For the pathogenesis of MS, both genetic (HLA II genes) and environmental factors (i.e., vitamin D levels, smoking) contribute to disease susceptibility (126, 127). The autoimmune process involves both the gray and white matter, thus explaining the cognition alterations often found in MS patients. The destruction patterns in the MS plaque can include CD4^+^ T cells, which play a key role in the immune cascade activation, leading to tissue damage, cytotoxic attack mediated by CD8^+^ T cells and macrophages, as well as a humoral-mediated destruction of the myelin structure through the local production of antibodies with consequent complement activation (128).

The most studied model of MS in animals is EAE, in which autoimmune attack toward CNS is induced in susceptible strains of mice, through immunization with self-antigens derived from basic myelin protein. Autoreactive T cells traffic to the brain and to the spinal cord and damage the myelin structure of CNS, resulting in a chronic or relapsing-remitting paralysis (depending on the antigen used for immunization and the strain of mice). In the inflammatory lesions, and increased secretion of Th1 cytokines has been detected, whereas Th2 cytokines typically associate with recovery and protection from EAE (129).

Recent findings have shown that the immunomodulatory effects of leptin are involved in the induction and progression of EAE, a mouse model of MS (129, 130). The *ob/ob* mice do not develop EAE, a condition associated with increased IL-4 production and a decrease in IFN-γ secretion by T cells upon antigen-specific stimulation. On the contrary exogenous leptin treatment renders *ob/ob* mice susceptible to EAE development, by increasing pro-inflammatory cytokine production (129).

Leptin neutralization in EAE-affected WT mice inhibits T cell functions, significantly delays disease progression with the final effect of improvement of the clinical symptoms (131).

In addition, high leptin levels have been reported also in active inflammatory lesions of the CNS of MS patients (132) and in the sera of MS patients treated with IFN-β before the relapses (133). In humans, it has been also shown that leptin production was significantly increased in both serum and CSF of naïve-to-therapy RRMS patients and its levels inversely correlated with frequency of Treg cells (134).

Recently the adipose tissue, through leptin production, has been shown to play a pivotal role in the survival of autoantigen-specific CD4^+^ T cells *in vivo*, through the activation of mTOR pathway and the induction of Bcl-2 (direct mechanism) and through the reduction of a series of cytokines, whose production is important for autoreactive cell survival (IL-6, IL-15, IL-21, and GM-CSF) (indirect mechanism) (135). Recently, it has been demonstrated that Tregs proliferation is impaired in RRMS patients because of altered IL-2 secretion and IL-2R-signal transducer and activator of transcription 5 (STAT5) signaling. These results suggest the presence of an altered metabolic control accounting for the progressive loss of Treg cells in autoimmune disease (136). The expression of LepR has been found to be significantly higher in CD8^+^ T cells and monocytes from MS patients in relapse phase than those observed in patients in remission (or in healthy controls). Moreover, relapsing patients display high levels of phospho-signal transducer and activator of transcription-3 (P-STAT-3) and low expression of suppressor of cytokine signaling-3 (SOCS-3) and exogenous leptin treatment sustains STAT3 phosphorylation only in the monocytes from relapsing patients, suggesting that LepR might play a role in the modulation of clinical relapses during MS (137). A recent report has shown that obesity and high leptin levels at age of 18 associate with a greater than twofold increased risk of MS development (138). Comparable epidemiological evidence has suggested that subjects whose BMI exceeded 27 kg/m^2^, had a twofold increased risk to develop MS in a cohort of Swedish population (139). In addition, Hedström et al. have also shown that a possible interactions between BMI and MS could be associated to HLA genotype (DRB1*15 and absence of A*02) (140). The authors hypothesized that one possible explaination for this association is the lower levels of 25-hydroxy vitamin D discovered in obese patients compared to non-obese subjects, since 25-hydroxyvitamin D levels have been shown to be protective from MS development (126). The finding of an interaction between obesity and HLA genotype with regard to MS supports the hypothesis that the Th1-promoting effects of obesity increase the risk of developing MS, in particular among subjects with a genetic susceptibility to the disease. In this context, prevention of obesity in adolescents may therefore play a role in reducing the risk to develop MS, above all in subjects with a genetic susceptibility.

Importantly, a dycothomous role of leptin on the CNS has recently emerged. While leptin can participate in the immune-mediated attack to myelin, new evidence suggests that leptin may have differential effects on myelination and neural cell survival, acting as a neurotrophic factor (141, 142). Indeed, the brain weight of *ob/ob* and *db/db* mice is significantly reduced and these mice express synaptic and glial proteins with immature characteristics. They also display elevated expression of growth-associated protein in the neocortex and hippocampus, and decreased expression of syntaxin-1, synaptosomal-associated protein-25, and synaptobrevin (141, 142). Leptin deficiency also associates with a decreased expression of myelin basic protein (MBP) and/or proteolipid protein (PLP) in the neocortex, hippocampus. By contrast, recombinant leptin administration is able to revert this phenotype, by increasing brain weight, restoring a proper proteic asset, and sustaining the overall locomotor activity of these animals, thus suggesting that leptin requirement is essential for the physiological development of the nervous system.

## Role of Central Leptin Signaling in the Control of Immune System

Despite the studies previously mentioned focused mainly on the role of leptin in the modulation of peripheral immune cells functions (106, 110, 112), only recently it has become increasingly evident that the leptin signaling at the central level (CNS) is itself able to directly modulate immune system.

Recent papers have suggested that leptin deficiency reduced renal macrophage infiltration in a model of unilateral ureteral obstruction (UOO) (143). Interestingly, central leptin administration in *ob/ob* mice was able to revert this condition. The authors also showed that co-treatment with a melanocortin-3 receptor (MC3R)/melanocortin-4 receptor (MC4R) antagonist, blunted leptin effects, thus suggesting that leptin increases renal macrophage infiltration through the activation of the central melanocortin system (143).

In addition, intracerebroventricular leptin injection was sufficient to prevent the alteration of B-cell development in the bone marrow of fasted mice (characterized by altered balance between immature and mature B-cells), thus providing again the *in vivo* evidence for the role of central leptin signaling in B-cell development (144). Other studies have shown that leptin-deficient mice showed an increased susceptibility to sepsis and mortality, due to an impaired recruitment and function of neutrophils. On the contrary, the treatment with leptin exclusively at the intracerebral level, improved the survival, and the risk of infection of these mice, again suggesting the importance of the central leptin signaling in the modulation of immune functions (145).

## Concluding Remarks

It is becoming increasingly evident that there is a dense and intricate relationship between the immune and nervous system (146). This type of interaction is explicated through the production of molecules (cytokines, hormones, and peptides) from the CNS and through the activation of afferent and efferent neurological pathways in lymphoid organs, with both immuno-suppressive and immuno-stimulating effects. On the other hand, also the cytokines themselves are able to communicate with the CNS and ensure the passage of specific signals and information from the periphery to the brain. In this context, leptin represents a key factor linking immune system, metabolism, and CNS functions.

The comprehensive and extensive understanding of the mechanisms underlying the interaction between the CNS and immune systems, may allow the modulation of certain brain functions as a possible clinic therapeutic approaches for immune-mediated diseases.

Recent reports have shown that caloric restriction (CR) (associates with a fall in plasma leptin levels) can significantly increase the overall survival in several experimental animal models of autoimmune diseases (147). More specifically, CR has anti-inflammatory, antioxidant, and neuroprotective effects that could be instrumental for an improvement of clinical outcomes in MS, since this regimen is able to impair pathological proliferation of autoreactive cells and pro-inflammatory cytokine production in EAE (147).

Although several evidence has suggested that diet may alter the course and progression of autoimmune diseases (i.e., the case of MS), only few randomized studies of dietary alterations in MS have been conducted so far, and none of them seem to include CR regimen.

The future goal of the research will be to assess how and whether CR might actually be a useful therapeutical approach for MS. A careful monitoring of patients could in fact ensure beneficial effects in terms of reduction of inflammation and at the same time could determine the improvement of other clinical parameters such as insulin sensitivity, low-density lipoprotein, cholesterol, blood pressure, which would be crucial for the disease amelioration.

## Conflict of Interest Statement

The authors declare that the research was conducted in the absence of any commercial or financial relationships that could be construed as a potential conflict of interest.

## References

[1] Besedovsky HO Del Rey AE Sorkin E Da Prada M Burri R Honegger C The immune response evokes changes in brain noradrenergic neurons. Science (1983) 221:564–5 10.1126/science.6867729 6867729

[2] Blalock JE A molecular basis for bidirectional communication between the immune and neuroendocrine systems. Physiol Rev (1989) 69:1–27 2536183 10.1152/physrev.1989.69.1.1

[3] Carlson SL Felten DL Livnat S Felten SY Alterations of monoamines in specific central autonomic nuclei following immunization in mice. Brain Behav Immun (1987) 1:52–63 10.1016/0889-1591(87)90006-7 3451782

[4] Sundar SK Cierpial MA Kamaraju LS Long S Hsieh S Lorenz C Human immunodeficiency virus glycoprotein (gp120) infused into rat brain induces interleukin 1 to elevate pituitary-adrenal activity and decrease peripheral cellular immune responses. Proc Natl Acad Sci U S A (1991) 88:11246–50 10.1073/pnas.88.24.11246 1662389 PMC53111

[5] Watkins LR Maier SF Goehler LE Cytokine-to-brain communication: a review and analysis of alternative mechanisms. Life Sci (1995) 57:1011–26 10.1016/0024-3205(95)02047-M 7658909

[6] Dantzer R Innate immunity at the forefront of psychoneuroimmunology. Brain Behav Immun (2004) 18:1–6 10.1016/j.bbi.2003.09.008 14651940

[7] Carr DJJ Blalock JE Neuropeptide hormones and receptors common to the immune and neuroendocrine systems: bidirectional pathway of intersystem communication. In: Ader R Felten DL Cohen N, editors. Psychoneuroimmunology. (Vol. 2), San Diego, CA: Academic Press (1991). p. 573–88

[8] Blalock JE Shared ligands and receptors as a molecular mechanism for communication between the immune and neuroendocrine systems. Ann N Y Acad Sci (1994) 741:292–8 10.1111/j.1749-6632.1994.tb23112.x 7825817

[9] Harbour DV Smith EM Blalock JE Splenic lymphocyte production of an endorphin during endotoxic shock. Brain Behav Immun (1987) 1:123–33 10.1016/0889-1591(87)90015-8 3330673

[10] Lolait SJ Lim AT Toh BH Funder JW Immunoreactive beta-endorphin in a subpopulation of mouse spleen macrophages. J Clin Invest (1984) 73:277–80 10.1172/JCI111203 6317717 PMC425012

[11] Engler KL Rudd ML Ryan JJ Stewart JK Fisher-Stenger K Autocrine actions of macrophage-derived catecholamines on interleukin-1h. J Neuroimmunol (2005) 160:87–91 10.1016/j.jneuroim.2004.11.005 15710461

[12] Hattori N Inagaki C Immunological aspects of human growth hormone and prolactin. Domest Anim Endocrinol (1998) 15:371–5 10.1016/S0739-7240(98)00019-8 9785041

[13] Schulte-Herbruggen O Nassenstein C Lommatzsch M Quarcoo D Renz H Braun A Tumor necrosis factor-α and interleukin-6 regulates secretion of brain-derived neurotrophic factor in human monocytes. J Neuroimmunol (2005) 160:204–9 10.1016/j.jneuroim.2004.10.026 15710474

[14] Wrona D Neural-immune interactions: an integrative view of the bidirectional relationship between the brain and immune systems. J Neuroimmunol (2006) 172:38–58 10.1016/j.jneuroim.2005.10.017 16375977

[15] Rivest S Molecular insights on the cerebral innate immune system. Brain Behav Immun (2003) 17:13–9 10.1016/S0889-1591(02)00055-7 12615045

[16] Ousman SS Kubes P Immune surveillance in the central nervous system. Nat Neurosci (2012) 15:1096–101 10.1038/nn.3161 22837040 PMC7097282

[17] Raivich G Like cops on the beat: the active role of resting microglia. Trends Neurosci (2005) 28:571–3 10.1016/j.tins.2005.09.001 16165228

[18] Hickey WF Basic principles of immunological surveillance of the normal central nervous system. Glia (2001) 36:118–24 10.1002/glia.1101 11596120

[19] Hayes GM Woodroofe MN Cuzner ML Microglia are the major cell type expressing MHC class II in human white matter. J Neurol Sci (1987) 80:25–37 10.1016/0022-510X(87)90218-8 3302117

[20] Nimmerjahn A Kirchhoff F Helmchen F Resting microglial cells are highly dynamic surveillants of brain parenchyma in vivo. Science (2005) 308:1314–8 10.1126/science.1110647 15831717

[21] Akiyama H Itagaki S McGeer PL Major histocompatibility complex antigen expression on rat microglia following epidural kainic acid lesions. J Neurosci Res (1988) 20:147–57 10.1002/jnr.490200202 3172275

[22] Akiyama H McGeer PL Brain microglia constitutively express β-2 integrins. J Neuroimmunol (1990) 30:81–93 10.1016/0165-5728(90)90055-R 1977769

[23] Shrikant P Benveniste EN The central nervous system as an immunocompetent organ: role of glial cells in antigen presentation. J Immunol (1996) 157:1819–22 8757296

[24] Ford AL Foulcher E Lemckert FA Sedgwick JD Microglia induce CD4^+^ T lymphocyte final effector function and death. J Exp Med (1996) 184:1737–45 10.1084/jem.184.5.1737 8920862 PMC2192872

[25] Hickey WF Hsu BL Kimura H T-lymphocyte entry into the central nervous system. J Neurosci Res (1991) 28:254–60 10.1002/jnr.490280213 2033653

[26] Ransohoff RM Kivisäkk P Kidd G Three or more routes for leukocyte migration into the central nervous system. Nat Rev Immunol (2003) 3:569–81 10.1038/nri1130 12876559

[27] Kivisäkk P Mahad DJ Callahan MK Trebst C Tucky B Wei T Human cerebrospinal fluid central memory CD4+ T cells: evidence for trafficking through choroid plexus and meninges via P-selectin. Proc Natl Acad Sci U S A (2003) 100:8389–94 10.1073/pnas.1433000100 12829791 PMC166239

[28] Kivisäkk P Trebst C Liu Z Tucky BH Sørensen TL Rudick RA T-cells in the cerebrospinal fluid express a similar repertoire of inflammatory chemokine receptors in the absence or presence of CNS inflammation: implications for CNS trafficking. Clin Exp Immunol (2002) 129:510–8 10.1046/j.1365-2249.2002.01947.x 12197893 PMC1906480

[29] Svenningsson A Andersen O Edsbagge M Stemme S Lymphocyte phenotype and subset distribution in normal cerebrospinal fluid. J Neuroimmunol (1995) 63:39–46 10.1016/0165-5728(95)00126-3 8557823

[30] Carrithers MD Visintin I Viret C Janeway CS Jr Role of genetic background in P selectin-dependent immune surveillance of the central nervous system. J Neuroimmunol (2002) 129:51–7 10.1016/S0165-5728(02)00172-8 12161020

[31] Loeffler C Dietz K Schleich A Schlaszus H Stoll M Meyermann R Immune surveillance of the normal human CNS takes place in dependence of the locoregional blood-brain barrier configuration and is mainly performed by CD3^+^/CD8^+^ lymphocytes. Neuropathology (2011) 31:230–8 10.1111/j.1440-1789.2010.01167.x 21092063

[32] Tracey KJ Physiology and immunology of the cholinergic antiinflammatory pathway. J Clin Invest (2007) 117:289–96 10.1172/JCI30555 17273548 PMC1783813

[33] Wang H Yu M Ochani M Amella CA Tanovic M Susarla S Nicotinic acetylcholine receptor alpha7 subunit is an essential regulator of inflammation. Nature (2003) 42:384–8 10.1038/nature01339 12508119

[34] Rosas-Ballina M Olofsson PS Ochani M Valdés-Ferrer SI Levine YA Reardon C Acetylcholine-synthesizing T cells relay neural signals in a vagus nerve circuit. Science (2011) 334:98–101 10.1126/science.1209985 21921156 PMC4548937

[35] Wang H Liao H Ochani M Justiniani M Lin X Yang L Cholinergic agonists inhibit HMGB1 release and improve survival in experimental sepsis. Nat Med (2004) 10:1216–21 10.1038/nm1124 15502843

[36] Saeed RW Varma S Peng-Nemeroff T Sherry B Balakhaneh D Huston J Cholinergic stimulation blocks endothelial cell activation and leukocyte recruitment during inflammation. J Exp Med (2005) 201:1113–23 10.1084/jem.20040463 15809354 PMC2213139

[37] van Maanen MA Lebre MC van der Poll T LaRosa GJ Elbaum D Vervoordeldonk MJ Stimulation of nicotinic acetylcholine receptors attenuates collagen-induced arthritis in mice. Arthritis Rheum (2009) 60:114–22 10.1002/art.24177 19116908

[38] van Maanen MA Vervoordeldonk MJ Tak PP The cholinergic anti-inflammatory pathway: towards innovative treatment of rheumatoid arthritis. Nat Rev Rheumatol (2009) 5:229–32 10.1038/nrrheum.2009.31 19337288

[39] Holguin F Téllez-Rojo MM Lazo M Mannino D Schwartz J Hernández M Cardiac autonomic changes associated with fish oil vs soy oil supplementation in the elderly. Chest (2005) 127:1102–7 10.1378/chest.127.4.1102 15821181

[40] Facchini M Malfatto G Sala L Silvestri G Fontana P Lafortuna C Changes of autonomic cardiac profile after a 3-week integrated body weight reduction program in severely obese patients. J Endocrinol Invest (2003) 26:138–42 12739741 10.1007/BF03345142

[41] Madden KS Moynihan JA Brenner GJ Felten SY Felten DL Livnat S Sympathetic nervous system modulation of the immune system III. Alterations in T and B cell proliferation and differentiation in vitro following chemical sympathectomy. J Neuroimmunol (1994) 49:77–87 10.1016/0165-5728(94)90183-X 8294564

[42] Madden KS Catecholamines, sympathetic innervation, and immunity. Brain Behav Immun (2003) 17:S5–10 10.1016/S0889-1591(02)00059-4 12615180

[43] Benschop RJ Rodriguez-Feuerhahn M Schedlowski M Catecholamine-induced leukocytosis: early observations, current research, and future directions. Brain Behav Immun (1996) 10:77–91 10.1006/brbi.1996.0009 8811932

[44] Saha B Mondal AC Majumder J Basu S Dasgupta PS Physiological concentrations of dopamine inhibit the proliferation and cytotoxicity of human CD4^+^ and CD8^+^ T cells in vitro: a receptor mediated mechanism. Neuroimmunomodulation (2001) 9:23–33 10.1159/000049004 11435749

[45] Carr L Tucker A Fernandez-Botran R In vivo administration of L-DOPA or dopamine decreases the number of splenic IFN-γ producing cells. J Neuroimmunol (2003) 137:87–93 10.1016/S0165-5728(03)00047-X 12667651

[46] Kelley K Growth hormone, lymphocytes and macrophages. Biochem Pharmacol (1989) 38:705–13 10.1016/0006-2952(89)90222-0 2649106

[47] Dorshkind K Horseman ND The roles of prolactin, growth hormone, insulin-like growth factor-I, and thyroid hormones in lymphocyte development and function: insights from genetic models of hormone and hormone receptor deficiency. Endocr Rev (2000) 21:292–312 10.1210/er.21.3.292 10857555

[48] Carreno PC Sacedon R Jimenez E Vincente A Zapata AG Prolactin affects both survival and differentiation of T-cell progenitors. J Neuroimmunol (2005) 160:135–45 10.1016/j.jneuroim.2004.11.008 15710466

[49] Esquifino AI Arce A Alvarez MP Chacon F Brown-Borg H Bartke A Differential effects of light/dark recombinant human prolactin administration on the submaxillary lymph nodes and spleen activity of adult male mice. Neuroimmunomodulation (2004) 11:119–26 10.1159/000075321 14758058

[50] Wyllie AH Glucocorticoid induced thymocytes apoptosis is associated with endogenous endonuclease activation. Nature (1980) 284:555–6 10.1038/284555a0 6245367

[51] Ucker DS Cytotoxic T lymphocytes and glucocorticoids activate an endogenous suicide process in target cells. Nature (1987) 327:62–4 10.1038/327062a0 3494953

[52] Sternberg EM Neuroendocrine regulation of autoimmune/inflammatory disease. J Endocrinol (2001) 169:429–35 10.1677/joe.0.1690429 11375112

[53] Eskandari F Sternberg EM Neural-immune interactions in health and disease. Ann N Y Acad Sci (2002) 966:20–7 10.1111/j.1749-6632.2002.tb04198.x 12114255

[54] Felten DL Felten SY Carlson SL Olschowka JA Livnat S Noradrenergic and peptidergic innervation of lymphoid tissue. J Immunol (1985) 135:755–65 2861231

[55] Bellinger DL Lorton D Romano T Olschowka JA Felten SY Felten DL Neuropeptide innervation of lymphoid organs. Ann N Y Acad Sci (1990) 594:17–33 10.1111/j.1749-6632.1990.tb40464.x 2165757

[56] Matzinger P An innate sense of danger. Ann N Y Acad Sci (2002) 961:341–2 10.1111/j.1749-6632.2002.tb03118.x 12081934

[57] Bianchi ME DAMPs, PAMPs and alarmins: all we need to know about danger. J Leukoc Biol (2007) 81:1–5 10.1189/jlb.0306164 17032697

[58] Levine JD Khasar SG Green PG Neurogenic inflammation and arthritis. Ann N Y Acad Sci (2006) 1069:155–67 10.1196/annals.1351.014 16855143

[59] Levite M Chowers Y Ganor Y Besser M Hershkovits R Cahalon L Dopamine interacts directly with its D3 and D2 receptors on normal human T cells, and activates beta1 integrin function. Eur J Immunol (2001) 12:3504–12 10.1002/1521-4141(200112)31:12<3504::AID-IMMU3504>3.0.CO;2-F 11745370

[60] Levite M Hart I Immunotherapy for epilepsy. Expert Rev Neurother (2002) 2:809–14 10.1586/14737175.2.6.809 19810914

[61] Levite M Fleidervish IA Schwarz A Pelled D Futerman AH Autoantibodies to the glutamate receptor kill neurons via activation of the receptor ion channel. J Autoimmun (1999) 13:61–72 10.1006/jaut.1999.0301 10441169

[62] Ganea D Delgado M The neuropeptides VIP/PACAP and T cells: inhibitors or activators? Curr Pharm Des (2003) 9:997–1004 10.2174/1381612033455116 12678866

[63] Ganea D Rodriquez R Delgado M Vasoactive intestinal peptide and pituitary adenylate cyclase-activating polypeptide: players in innate and adaptive immunity. Cell Mol Biol (2003) 49:127–42 12887096

[64] Delgado M Gonzalez-Rey E Ganea D VIP/PACAP preferentially attract Th2 effectors through differential regulation of chemokine production by dendritic cells. FASEB J (2004) 18:1453–5 15231725 10.1096/fj.04-1548fje

[65] Delgado M Pozo D Ganea D The significance of vasoactive intestinal peptide in immunomodulation. Pharmacol Rev (2004) 56:249–90 10.1124/pr.56.2.7 15169929

[66] De la Fuente M Bernaez I Del Rio M Hernanz A Stimulation of murine peritoneal macrophage functions by neuropeptide Y and peptide YY. Involvement of protein kinase C. Immunology (1993) 80:259–65 8262554 PMC1422192

[67] Puerto M Guayerbas N Alvarez P De la Fuente M Modulation of neuropeptide Y and norepinephrine on several leukocyte functions in adult, old and very old mice. J Neuroimmunol (2005) 165:33–40 10.1016/j.jneuroim.2005.03.021 16005734

[68] Feistritzer C Clausen J Sturn DH Djanani A Gunsilius E Wiedermann CJ Natural killer cell functions mediated by the neuropeptide substance P. Regul Pept (2003) 116:119–26 10.1016/S0167-0115(03)00193-9 14599723

[69] Jing H Yen JH Ganea D A novel signaling pathway mediates the inhibition of CCL3/4 expression by prostaglandin E2. J Biol Chem (2004) 279:55176–86 10.1074/jbc.M409816200 15498767

[70] Gropp E Shanabrough M Borok E Xu AW Janoschek R Buch T Agouti-related peptide-expressing neurons are mandatory for feeding. Nat Neurosci (2005) 8:1289–91 10.1038/nn1548 16158063

[71] Dietrich MO Bober J Ferreira JG Tellez LA Mineur YS Souza DO AgRP neurons regulate the development of dopamine neuronal plasticity and non food-associated behaviors. Nat Neurosci (2012) 5:1108–10 10.1038/nn.3147 22729177 PMC3411867

[72] Dietrich MO Antunes C Geliang G Liu ZW Borok E Nie Y Agrp neurons mediate Sirt1’s action on the melanocortin system and energy balance: roles for Sirt1 in neuronal firing and synaptic plasticity. J Neurosci (2010) 30:11815–25 10.1523/JNEUROSCI.2234-10.2010 20810901 PMC2965459

[73] Matarese G Procaccini C Menale C Kim JG Kim JD Diano S Hunger-promoting hypothalamic neurons modulate effector and regulatory T-cell responses. Proc Natl Acad Sci U S A (2013) 110:6193–8 10.1073/pnas.1210644110 23530205 PMC3625304

[74] Joly-Amado A Denis RG Castel J Lacombe A Cansell C Rouch C Hypothalamic AgRP-neurons control peripheral substrate utilization and nutrient partitioning. EMBO J (2012) 31:4276–88 10.1038/emboj.2012.250 22990237 PMC3501217

[75] Gutierrez EG Banks WA Kastin AJ Murine tumor necrosis factor alpha is transported from blood to brain in the mouse. J Neuroimmunol (1993) 47:169–76 10.1016/0165-5728(93)90027-V 8370768

[76] Gutierrez EG Banks WA Kastin AJ Blood-borne interleukin-1 receptor antagonist crosses the blood-brain barrier. J Neuroimmunol (1994) 55:153–60 10.1016/0165-5728(94)90005-1 7829665

[77] Weller RO Engelhardt B Phillips MM Lymphocyte targeting of the central nervous system: a review of afferent and efferent CNS-immune pathways. Brain Pathol (1996) 6:275–88 10.1111/j.1750-3639.1996.tb00855.x 8864284

[78] Goehler LE Gaykema RP Hansen MK Anderson K Maier SF Watkins LR Vagal immune-to-brain communication: a visceral chemosensory pathway. Auton Neurosci (2000) 85:49–59 10.1016/S1566-0702(00)00219-8 11189026

[79] Marvel FA Chen C Badr N Gaykema RPA Goehler LE Reversible inactivation of the dorsal vagal complex blocks lipopolysaccharide-induced social withdrawal and c-Fos expression in central autonomic nuclei. Brain Behav Immun (2004) 18:123–34 10.1016/j.bbi.2003.09.004 14759590

[80] Munck A Guyre PM Glucocorticoids and immune function. In: Ader R Felten DL Cohen N, editors. Psychoneuroimmunology. (Vol. 2), San Diego, CA: Academic Press (1991). p. 447–74

[81] Besedovsky HO Sorkin E Keller M Muller J Changes in blood hormone levels during the immune response. Proc Soc Exp Biol Med (1975) 150:466–70 10.3181/00379727-150-39057 1208563

[82] Besedovsky HO Del Rey A Sorkin E Dinarello CA Immunoregulatory feedback between interleukin-1 and glucocorticoid hormones. Science (1986) 233:652–4 10.1126/science.3014662 3014662

[83] Schettini G Interleukin-1 in the neuroendocrine system from gene to function. Prog Neuroendocrinol Immunol (1990) 3:157–66

[84] Berkenbosch F Oers J van Del Rey A Tilders F Besedovsky H Corticotropin-releasing factor-producing neurons in the rat activated by interleukin-1. Science (1987) 238:524–6 10.1126/science.2443979 2443979

[85] Sapolsky R Rivier C Yamamoto G Plotsky P Vale W Interleukin-1 stimulates the secretion of hypothalamic corticotropin-releasing factor. Science (1987) 238:522–4 10.1126/science.2821621 2821621

[86] Fontana A Grob PJ Astrocyte-derived interleukin-1 like factors. Lymphokine Res (1984) 3:11–6 6088903

[87] Giulian D Baker TJ Shih LC Lachman LB Interleukin-1 of the central nervous system is produced by ameboid microglia. J Exp Med (1986) 164:594–604 10.1084/jem.164.2.594 3487617 PMC2188228

[88] Breder CD Dinarello CA Saper CB Interleukin-1 immunoreactive innervation of the human hypothalamus. Science (1988) 240:321–4 10.1126/science.3258444 3258444

[89] Cunninham ET De Souza EB Interleukin 1 receptors in the brain and endocrine tissues. Immunol Today (1993) 14:171–6 8499077 10.1016/0167-5699(93)90281-o

[90] Koenig JI Snow K Clark BD Toni R Cannon JG Shaw AR Intrinsic pituitary interleukin-1 beta is induced by bacteria. Endocrinology (1990) 126:3053–8 10.1210/endo-126-6-3053 2190803

[91] Gatti S Bartfai T Induction of tumor necrosis factor-alpha mRNA in the brain after peripheral endotoxin treatment: comparison with interleukin-1 family and interleukin-6. Brain Res (1993) 624:291–4 10.1016/0006-8993(93)90090-A 8252403

[92] Ching S He L Lai W Quan N IL-1 type receptor plays a key role in mediating the recruitment of leukocytes into the central nervous system. Brain Behav Immun (2005) 19:127–37 10.1016/j.bbi.2004.06.001 15664785

[93] Schiffenbauer J Streit WJ Butfiloski E LaBow M Edwards C Moldawer LL The induction of EAE is only partially dependent on TNF receptor signaling but require the IL-1 type I receptor. Clin Immunol (2000) 95:117–23 10.1006/clim.2000.4851 10779405

[94] Bernardes-Silva M Anthony DC Issekutz AC Perry VH Recruitment of neutrophils across the blood-brain barrier: the role of E- and P-selectins. J Cereb Blood Flow Metab (2001) 21:1115–24 10.1097/00004647-200109000-00009 11524616

[95] Sarder M Saito H Abe K Interleukin-2 promotes survival and neurite extension of cultured neurons from fetal rat brain. Brain Res (1993) 625:347–50 10.1016/0006-8993(93)91080-C 8275319

[96] Beck RD Jr King MA Huang Z Petitto JM Alterations in septohippocampal cholinergic neurons resulting from interleukin-2 gene knockout. Brain Res (2002) 955:16–23 10.1016/S0006-8993(02)03295-X 12419517

[97] Beck RD Jr King MA Ha GK Cushman JD Huang Z Petittov JM IL-2 deficiency results in altered septal and hippocampal cytoarchitecture: relation to development and neurotrophins. J Neuroimmunol (2005) 160:146–53 10.1016/j.jneuroim.2004.11.006 15710467

[98] Friedman JM Halaas JL Leptin and the regulation of body weight in mammals. Nature (1998) 395:763–70 10.1038/27376 9796811

[99] Procaccini C Jirillo E Matarese G Leptin as an immunomodulator. Mol Aspects Med (2012) 33:35–45 10.1016/j.mam.2011.10.012 22040697

[100] Procaccini C De Rosa V Galgani M Carbone F Cassano S Greco D Leptin-induced mTOR activation defines a specific molecular and transcriptional signature controlling CD4+ effector T cell responses. J Immunol (2012) 189:2941–53 10.4049/jimmunol.1200935 22904304

[101] Procaccini C Galgani M De Rosa V Matarese G Intracellular metabolic pathways control immune tolerance. Trends Immunol (2012) 33:1–7 22075206 10.1016/j.it.2011.09.002

[102] Chehab FF Lim ME Lu R Correction of the sterility defect in homozygous obese female mice by treatment with the human recombinant leptin. Nat Genet (1996) 12:318–20 10.1038/ng0396-318 8589726

[103] Bennett BD Solar GP Yuan JQ Mathias J Thomas GR Matthews W A role for leptin and its cognate receptor in hematopoiesis. Curr Biol (1996) 6:1170–80 10.1016/S0960-9822(02)70684-2 8805376

[104] Sierra-Honigmann MR Nath AK Murakami C García-Cardeña G Papapetropoulos A Sessa WC Biological action of leptin as an angiogenic factor. Science (1998) 28:1683–6 10.1126/science.281.5383.1683 9733517

[105] Ducy P Amling M Takeda S Priemel M Schilling AF Beil FT Leptin inhibits bone formation through a hypothalamic relay: a central control of bone mass. Cell (2000) 100:197–207 10.1016/S0092-8674(00)81558-5 10660043

[106] Lord GM Matarese G Howard JK Baker RJ Bloom SR Lechler RI Leptin modulates the T-cell immune response and reverses starvation-induced immunosuppression. Nature (1998) 394:897–901 10.1038/29795 9732873

[107] Sánchez-Margalet V Martín-Romero C Santos-Alvarez J Goberna R Najib S Gonzalez-Yanes C Role of leptin as an immunomodulator of blood mononuclear cells: mechanisms of action. Clin Exp Immunol (2003) 133:11–9 10.1046/j.1365-2249.2003.02190.x 12823272 PMC1808745

[108] Mancuso P Gottschalk A Phare SM Peters-Golden M Lukacs NW Huffnagle GB Leptin-deficient mice exhibit impaired host defense in Gram-negative pneumonia. J Immunol (2002) 168:4018–24 11937559 10.4049/jimmunol.168.8.4018

[109] Loffreda S Yang SQ Lin HZ Karp CL Brengman ML Wang DJ Leptin regulates proinflammatory immune responses. FASEB J (1998) 1:57–65 9438411

[110] Gainsford T Willson TA Metcalf D Handman E McFarlane C Ng A Leptin can induce proliferation, differentiation, and functional activation of hemopoietic cells. Proc Natl Acad Sci U S A (1996) 93:14564–8 10.1073/pnas.93.25.14564 8962092 PMC26173

[111] Caldefie-Chezet F Poulin A Tridon A Sion B Vasson MP Leptin: a potential regulator of polymorphonuclear neutrophil bactericidal action? J Leukoc Biol (2001) 69:414–8 11261788

[112] Caldefie-Chezet F Poulin A Vasson MP Leptin regulates functional capacities of polymorphonuclear neutrophils. Free Radic Res (2003) 37:809–14 10.1080/1071576031000097526 14567439

[113] Tian Z Sun R Wei H Gao B Impaired natural killer (NK) cell activity in leptin receptor deficient mice: leptin as a critical regulator in NK cell development and activation. Biochem Biophys Res Commun (2002) 298:297–302 10.1016/S0006-291X(02)02462-2 12413939

[114] Howard JK Lord GM Matarese G Vendetti S Ghatei MA Ritter MA Leptin protects mice from starvation-induced lymphoid atrophy and increases thymic cellularity in ob/ob mice. J Clin Invest (1999) 104:1051–9 10.1172/JCI6762 10525043 PMC408574

[115] De Rosa V Procaccini C Calì G Pirozzi G Fontana S Zappacosta S A key role of leptin in the control of regulatory T cell proliferation. Immunity (2007) 26:241–55 10.1016/j.immuni.2007.01.011 17307705

[116] Procaccini C De Rosa V Galgani M Abanni L Calì G Porcellini A An oscillatory switch in mTOR kinase activity sets regulatory T cell responsiveness. Immunity (2010) 33:929–41 10.1016/j.immuni.2010.11.024 21145759 PMC3133602

[117] Fraser DA Thoen J Reseland JE Førre O Kjeldsen-Kragh J Decreased CD4^+^ lymphocyte activation and increased interleukin-4 production in peripheral blood of rheumatoid arthritis patients after acute starvation. Clin Rheumatol (1999) 18:394–401 10.1007/s100670050125 10524554

[118] Busso N So A Chobaz-Péclat V Morard C Martinez-Soria E Talabot-Ayer D Leptin signaling deficiency impairs humoral and cellular immune responses and attenuates experimental arthritis. J Immunol (2002) 168:875–82 11777985 10.4049/jimmunol.168.2.875

[119] Yu Y Liu Y Shi FD Zou H Matarese G La Cava A Cutting edge: leptin-induced RORγt expression in CD4^+^ T cells promotes Th17 responses in systemic lupus erythematosus. J Immunol (2013) 190:3054–8 10.4049/jimmunol.1203275 23447682 PMC3608794

[120] Tarzi RM Cook HT Jackson I Pusey CD Lord GM Leptin-deficient mice are protected from accelerated nephrotoxic nephritis. Am J Pathol (2004) 164:385–90 10.1016/S0002-9440(10)63128-8 14742244 PMC1602275

[121] Matarese G Sanna V Lechler RI Sarvetnick N Fontana S Zappacosta S Leptin accelerates autoimmune diabetes in female NOD mice. Diabetes (2002) 51:1356–61 10.2337/diabetes.51.5.1356 11978630

[122] Garcia-Gonzalez A Gonzalez-Lopez L Valera-Gonzalez IC Cardona-Muñoz EG Salazar-Paramo M González-Ortiz M Serum leptin levels in women with systemic lupus erythematosus. Rheumatol Int (2002) 22:138–41 10.1007/s00296-002-0216-9 12172951

[123] Matarese G Sanna V Di Giacomo A Lord GM Howard JK Bloom SR Leptin potentiates experimental autoimmune encephalomyelitis in SJL female mice and confers susceptibility to males. Eur J Immunol (2001) 31:1324–32 10.1002/1521-4141(200105)31:5<1324::AID-IMMU1324>3.0.CO;2-Y 11465089

[124] Williams KC Ulvestad E Hickey WF Immunology of multiple sclerosis. Clin Neurosci (1994) 2:229–45 7749893

[125] Lublin FD Reingold SC Defining the clinical course of multiple sclerosis: results of an international survey. National multiple sclerosis society (USA) advisory committee on clinical trials of new agents in multiple sclerosis. Neurology (1996) 46:907–11 10.1212/WNL.46.4.907 8780061

[126] Salzer J Hallmans G Nyström M Stenlund H Wadell G Sundström P Vitamin D as a protective factor in multiple sclerosis. Neurology (2012) 79:2140–5 10.1212/WNL.0b013e3182752ea8 23170011

[127] Hedström AK Sundqvist E Bäärnhielm M Nordin N Hillert J Kockum I Smoking and two human leukocyte antigen genes interact to increase the risk for multiple sclerosis. Brain (2011) 134:653–64 10.1093/brain/awq371 21303861

[128] Lassmann H Brück W Lucchinetti C Heterogeneity of multiple sclerosis pathogenesis: implications for diagnosis and therapy. Trends Mol Med (2001) 7:115–21 10.1016/S1471-4914(00)01909-2 11286782

[129] Matarese G Di Giacomo A Sanna V Lord GM Howard JK Di Tuoro A Requirement for leptin in the induction and progression of autoimmune encephalomyelitis. J Immunol (2001) 166:5909–16 11342605 10.4049/jimmunol.166.10.5909

[130] Sanna V Di Giacomo A La Cava A Lechler RI Fontana S Zappacosta S Leptin surge precedes onset of autoimmune encephalomyelitis and correlates with development of pathogenic T cell responses. J Clin Invest (2003) 111:241–50 10.1172/JCI200316721 12531880 PMC151876

[131] De Rosa V Procaccini C La Cava A Chieffi P Nicoletti GF Fontana S Leptin neutralization interferes with pathogenic T cell autoreactivity in autoimmune encephalomyelitis. J Clin Invest (2006) 116:447–55 10.1172/JCI26523 16410832 PMC1326145

[132] Lock C Hermans G Pedotti R Brendolan A Schadt E Garren H Gene-microarray analysis of multiple sclerosis lesions yields new targets validated in autoimmune encephalomyelitis. Nat Med (2002) 8:500–8 10.1038/nm0502-500 11984595

[133] Batocchi AP Rotondi M Caggiula M Frisullo G Odoardi F Nociti V Leptin as a marker of multiple sclerosis activity in patients treated with interferon-beta. J Neuroimmunol (2003) 139:150–4 10.1016/S0165-5728(03)00154-1 12799033

[134] Matarese G Carrieri PB La Cava A Perna F Sanna V De Rosa V Leptin increase in multiple sclerosis associates with reduced number of CD4^+^CD25^+^ regulatory T cells. Proc Natl Acad Sci U S A (2005) 102:5150–5 10.1073/pnas.0408995102 15788534 PMC555982

[135] Galgani M Procaccini C De Rosa V Carbone F Chieffi P La Cava A Leptin modulates the survival of autoreactive CD4^+^ T cells through the nutrient/energy-sensing mammalian target of rapamycin signaling pathway. J Immunol (2010) 185:7474–9 10.4049/jimmunol.1001674 21078910

[136] Carbone F De Rosa V Carrieri PB Montella S Bruzzese D Porcellini A Regulatory T cell proliferative potential is impaired in human autoimmune disease. Nat Med (2014) 20:69–74 10.1038/nm.3411 24317118

[137] Frisullo G Mirabella M Angelucci F Caggiula M Morosetti R Sancricca C The effect of disease activity on leptin, leptin receptor and suppressor of cytokine signalling-3 expression in relapsing-remitting multiple sclerosis. J Neuroimmunol (2007) 192:174–83 10.1016/j.jneuroim.2007.08.008 17904647

[138] Munger KL Chitnis T Ascherio A Body size and risk of MS in two cohorts of US women. Neurology (2009) 73:1543–50 10.1212/WNL.0b013e3181c0d6e0 19901245 PMC2777074

[139] Hedström AK Olsson T Alfredsson L High body mass index before age 20 is associated with increased risk for multiple sclerosis in both men and women. Mult Scler (2012) 18:1334–6 10.1177/1352458512436596 22328681

[140] Hedström AK Lima Bomfim I Barcellos L Gianfrancesco M Schaefer C Kockum I Interaction between adolescent obesity and HLA risk genes in the etiology of multiple sclerosis. Neurology (2014) 82:865–72 10.1212/WNL.0000000000000203 24500647 PMC3959752

[141] Valerio A Ghisi V Dossena M Tonello C Giordano A Frontini A Leptin increases axonal growth cone size in developing mouse cortical neurons by convergent signals inactivating glycogen synthase kinase-3 beta. J Biol Chem (2006) 281:12950–8 10.1074/jbc.M508691200 16522636

[142] Udagawa J Hashimoto R Suzuki H Hatta T Sotomaru Y Hioki K The role of leptin in the development of the cerebral cortex in mouse embryos. Endocrinology (2006) 147:647–58 10.1210/en.2005-0791 16282354

[143] Tanaka M Suganami T Sugita S Shimoda Y Kasahara M Aoe S Role of central leptin signaling in renal macrophage infiltration. Endocr J (2010) 57:61–72 10.1507/endocrj.K09E-296 19851035

[144] Tanaka M Suganami T Kim-Saijo M Toda C Tsuiji M Ochi K Role of central leptin signaling in the starvation-induced alteration of B-cell development. J Neurosci (2011) 31:8373–80 10.1523/JNEUROSCI.6562-10.2011 21653842 PMC6623333

[145] Tschöp J Nogueiras R Haas-Lockie S Kasten KR Castañeda TR Huber N CNS leptin action modulates immune response and survival in sepsis. J Neurosci (2010) 30:6036–47 10.1523/JNEUROSCI.4875-09.2010 20427662 PMC2868384

[146] Kamimura D Yamada M Harada M Sabharwal L Meng J Bando H The gateway theory: bridging neural and immune interactions in the CNS. Front Neurosci (2013) 7:204 10.3389/fnins.2013.00204 24194696 PMC3810779

[147] Piccio L Stark JL Cross AH Chronic calorie restriction attenuates experimental autoimmune encephalomyelitis. J Leukoc Biol (2008) 84:940–8 10.1189/jlb.0208133 18678605 PMC2638732

